# Discrete Element Method (DEM) Studies on Correcting the Particle Size Effect on the Shear Behaviors of Gravelly Soils

**DOI:** 10.3390/ma18092024

**Published:** 2025-04-29

**Authors:** Xiaolei Zhang, Zhenping Wu, Houyun Han, Yifeng Gao, Zhuofeng Li, Peng Xia

**Affiliations:** 1 Guangxi Pinglu Canal Constriction Co., Ltd., Nanning 535000, China; 13307724040@163.com; 2 China State Construction Port Engineering Group Ltd., Qingdao 266032, China; 18778876098@163.com (Z.W.); 15716558987@163.com (H.H.); 17707711143@163.com (Y.G.); 3 School of Civil Engineering and Architecture, Guangxi University, Nanning 530004, China; lzf@gxu.edu.cn; 4 State Key Laboratory of Precision Blasting, Hohai University, Nanjing 210024, China

**Keywords:** particle size effect, binary mixture, stiffness, laboratory test simulation, triaxial tests, granular mechanics, gravelly soils, mechanical behavior

## Abstract

The presence of overlarge gravel particles poses significant challenges for laboratory testing on prototype gravelly soils due to sample size limitations. To address this issue, replacement techniques, such as substituting overlarge particles with finer materials, offer practical solutions. However, the impact of these techniques on the mechanical behavior of gravelly soils, particularly shear strength and stiffness, remains poorly understood. This study aims to bridge this knowledge gap by investigating the particle size effect on the shear behaviors of binary mixtures using a series of Discrete Element Method (DEM) simulations. Updated scaling relations, based on Iai’s generalized scaling relations, were proposed to correct for particle size effects. DEM simulations, including drained triaxial tests and shear modulus measurements, were performed to validate the proposed law. The results indicate that the gravel replacement technique has a minor effect on peak shear strength but significantly reduces soil stiffness, especially at high gravel contents. The scaling relations effectively correct for the particle size effect, enabling the accurate prediction of shear behaviors of the prototype gravelly soils from those of the model gravelly soils. These validations demonstrate that for addressing the soil deformation problem instead of the stability problem in ultimate state, the developed scaling relations are highly effective for correcting the particle size effect. Based on the developed scaling relations, engineers can predict prototype-scale shear behaviors of gravelly soils with overlarge particles using scaled laboratory models, reducing reliance on costly large-scale equipment. Additionally, future studies, through both DEM simulations and laboratory experiments, are recommended to further validate and refine the proposed method across diverse soil conditions and loading scenarios, such as cyclic loadings.

## 1. Introduction

Gravelly soils, typically consisting of gravel and sand, are commonly encountered in natural deposits and are frequently utilized as construction materials [1,2,3,4,5]. In this study, ‘gravelly soils’ refer to mixtures of gravel (particles 4.75–75 mm) and sand, as classified by ASTM [6]. These materials exhibit soil-like mechanical responses under loading, including shear deformation and pore pressure generation. Such a wide range of gravel particle sizes poses a considerable challenge to laboratory element tests since the representative elementary volume and associated boundary effects necessitate a specimen diameter-to-maximum particle size ratio of at least 6 [6,7]. However, only a limited number of advanced geotechnical laboratories possess the large-capacity equipment required to accommodate specimens exceeding 300 mm in diameter [8]. To solve this obstacle, various methods have been proposed to handle the overlarge gravel particles in the prototype gravelly soils within the constraints of the existing testing apparatus. Among these, the replacement technique has gained widespread acceptance for assessing the mechanical responses of prototype gravelly soils with overlarge particles, which substitutes overlarge gravel particles with an equivalent mass of finer particles [9,10]. This treatment, however, raises critical questions regarding whether the mechanical responses of the scaled specimens accurately reflect those of the original specimens without any treatments of the overlarge gravel particles. Addressing these questions is essential for advancing our understanding of the particle size effect on the mechanical responses of gravelly soils with overlarge particles and for refining laboratory testing techniques on such materials. This issue is crucial for engineers to predict the prototype-scale shear behaviors of gravelly soils containing overlarge particles across small-to-large strain regimes using scaled laboratory models, eliminating the need for costly large-scale equipment.

Existing studies have reported that the utilization of sands as a substitute for overlarge gravel particles, while maintaining the specimen void ratio consistently, does not exert a significant influence on the mechanical responses of sand-like gravelly soils, which feature a gravel content (*GC*) of less than 40% (e.g., [8,11,12,13]). The effectiveness of the sand replacement techniques in gravelly soils with low *GC*s could be explained by the floating state of gravel particles within the sand–gravel mixtures [10]. But when the mixture skeleton evolves from gravel particles floating scattered in the sand matrix to sands filling the voids of the gravel matrix, typically around a threshold gravel content of 60% [14,15,16], such replacement techniques will significantly alter the mechanical responses of the prototype soils because the adoption of sand replacement techniques in gravelly soils with high *GC*s will change the mixture packing characteristics. For example, the shear strength, liquefaction resistance, and stiffness of gravelly soils with low *GC*s are greatly distinct from those with high *GC*s [2,17,18]. The above studies suggest the necessity to further investigate the particle size effect on the shear behaviors of gravelly soils, which is crucial for predicting the shear behaviors of gravelly soils containing overlarge particles in a cost-effective and reliable manner.

In addition to sand replacement, another approach involves substituting overlarge gravel particles with finer gravel particles. This method preserves the mixture’s skeleton characteristics and the packing density of the sand matrix while only reducing the size of gravel particles to accommodate experimental device limitations. Generally, such a technique provides more accurate predictions of the mechanical responses of prototype gravelly soils containing overlarge particles compared to sand replacement techniques. Nevertheless, some studies have shown that specimens with scaled gravel particles have dramatically different mechanical properties than the prototype ones, such as peak shear strength and critical state behaviors (e.g., [8,19,20]). Therefore, further investigation into the impact of gravel replacement techniques on the shear behaviors of gravelly soils with overlarge particles is essential, based on which the scaling relations between the mechanical responses of the model and prototype soils need to be established.

The Discrete Element Method (DEM), introduced by Cundall and Strack [21], serves as an effective tool for investigating the influence of particle size effects. Distinct from laboratory element testing, DEM simulations allow for the setting of idealized and consistent conditions, including the precise and homogeneous control of the specimen void ratio and gravel content throughout the whole specimen [22,23,24]. Hence, the DEM simulations were chosen as the target tool to fundamentally reveal the effect of particle size on the mechanical responses of gravelly soils with overlarge particles.

The present study aims to investigate the particle size effect on the shear behaviors of gravelly soils and provide insights into improving the laboratory testing methods for gravelly soils containing overlarge particles. First, the scaling relations for correcting the particle size effect were developed based on the generalized scaling relations (GSR) originally proposed by Iai [25]. Second, granular-scale numerical experiments employing DEM were systematically conducted to establish Hardin relationships for gravelly soils with typical *GC*s and particle size distributions, which enable the determination of key parameters for the developed scaling relations. Third, systematic DEM simulations involving drained triaxial tests and shear modulus tests were conducted to check the updated scaling relations. Based on the findings, recommendations were provided for performing laboratory element tests on gravelly soils containing overlarge particles using the gravel replacement method. The remainder of this paper is structured as follows: Section 2 details the developed scaling relations, Section 3 describes the DEM methodology, Section 4 present and discuss results, and Section 5 concludes with implications for practice and future research.

## 2. Scaling Relations for Correcting the Particle Size Effect

The Type I GSR introduced by Iai [25] is applicable for simulating cyclic mobility phenomena and can be adapted to address the particle size effect when predicting the mechanical responses of gravelly soils with overlarge particles. Within this scaling relation framework, the mechanical responses of both model soils and prototype soils must adhere to Rocha’s assumption [26], which necessitates the use of distinct scaling relations to describe stress and strain similarities [27]. Specifically, the stress scaling relation (*λ*_σ_) is typically derived using Equation (1), indicating that stress similarity depends on soil density and the length scaling relation (*λ*). Meanwhile, the strain scaling relation (*λ*_ε_) is determined through Equation (2), highlighting that strain similarity is influenced by the stiffness contrast between the model and prototype soils. All other mechanical property scaling relations are interconnected with three fundamental scaling relations: length (*λ*), density (*λ*_ρ_), and strain (*λ*_ε_). The relationships among these scaling relations for different mechanical properties are systematically outlined in Table 1 [25], which are all described by the above three fundamental scaling relations.

(1) $$\lambda_{\sigma }=\lambda \lambda_{\rho }$$

(2) $$\lambda_{\varepsilon }=\lambda /\left[{\left(V_{s}\right)}_{m}^{2}/{\left(V_{s}\right)}_{p}^{2}\right]$$

where the subscripts *p* and *m*, respectively, denote the prototype soils and model soils; *V*_s_ is the shear wave velocity of the soils.

One typical feature of the gravel replacement technique is the identical void ratio between the model soils and prototype soils. Prior research has demonstrated that gravelly soils with identical equivalent skeleton void ratios, despite varying gravel contents, will exhibit similar or comparable mechanical responses, including cyclic shear behavior [10,22,23], compression behaviors [16,28], critical behaviors [29], small-strain stiffness [30], etc. The setting of the same void ratio and gravel content but different maximum particle sizes between the model soils and prototype gravelly soils could be considered a stricter condition than the equivalent skeleton void ratio. Consequently, the mechanical responses of the prototype gravelly soils containing overlarge particles can, in principle, be accurately predicted from those soils with smaller gravel particles.

In the present study, the scaling relations for correcting the particle size effect were derived following the Type I GSR by Iai [25]. Under conditions of identical void ratios and soil grain specific gravities, the scaling relation for density *λ*_ρ_ equals 1. For laboratory element tests where the scaling relation for length *λ* is equal to 1, the scaling relation for stiffness *λ*_D_ is determined through Equation (3).

(3) $$\lambda_{D}=\lambda \lambda_{\rho }/\lambda_{\varepsilon }=1/\lambda_{\varepsilon }$$

Basically, the soil stiffness can be described by the famous Hardin equation (i.e., *G*_max_ = *AF*(e)(*σ*)*^n^*), from which the scaling relation presented in Equation (4) is derived [10]:

(4) $$\lambda_{D}=\frac{A_{p}F\left(e_{p}\right){\left(\sigma \right)}^{n_{p}}}{A_{m}F\left(e_{m}\right){\left(\sigma \right)}^{n_{m}}}$$

where *n* and *A* are material constants of the prepared granular soils; *F*(*e*) represents the void ratio function, which can be formulated as *F*(*e*) = (*a* − *e*)^2^/(1 + *e*), wherein a is a parameter tied to particle shape, taking a value of 2.17 for rounded particles and 2.97 for angular particles.; *σ* corresponds to the mean effective stress.

Existing research indicates that the parameter n for gravelly soils is influenced by *GC* [10,18] but remains relatively unaffected by the soil’s uniformity coefficient (*C*_u_) [18,31,32,33]. In contrast, *C*_u_ significantly impacts the parameter *A* [17,33]. Based on these findings, it is reasonable to assume that *n*_m_ = *n*_p_, enabling the reformulation of Equation (4) and Equation (3) into Equation (5) and Equation (6), respectively.

(5) $$\lambda_{D}=\frac{A_{p}F\left(e_{p}\right){\left(\sigma \right)}^{n_{p}}}{A_{m}F\left(e_{m}\right){\left(\sigma \right)}^{n_{m}}}=\frac{A_{p}}{A_{m}}=f\left(GC,\chi^{\prime }\right)$$

(6) $$\lambda_{\varepsilon }=1/f\left(GC,\chi^{\prime }\right)$$

where *χ*’ is the scaling relation for the maximum particle size, i.e., the maximum particle size ratio between the model and prototype gravelly soils; *f*(GC, *χ*’) is a function representing the scaling relation for parameter *A*, which is dependent on *GC* and *χ*’.

Drawing on the aforementioned derivations, the scaling relations for other mechanical properties can be established and are encapsulated in Table 1. In the updated scaling relations meant to correct the particle size effect, the strain scaling relation (*λ*_ε_) acts as the pivotal parameter, closely associated with the gravel content of the prototype soils and the variation in maximum particle size caused by the gravel replacement technique. Moving forward, DEM simulations were performed on small-strain shear modulus tests for fine-coarse binary mixtures with two distinct particle size distributions, which were used to simulate prototype and model gravelly soils. These simulations aimed to derive the essential parameters for establishing the scaling relations. Lastly, the accuracy of the updated scaling relations was assessed through DEM simulations of drained triaxial tests and shear modulus tests.

## 3. DEM Model and Simulation Arrangement

### 3.1. DEM Model Preparation and Parameters

**Figure 1:**
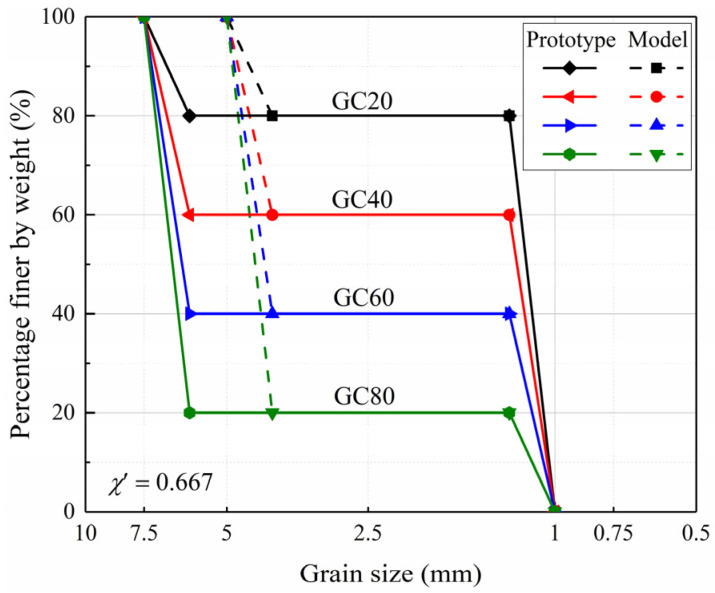
Particle size distribution curves of the prepared DEM models.

In this study, the well-recognized Discrete Element Method (DEM) software PFC^3D^ (Version PFC5.0) [34] was employed to carry out the simulations. For model preparation, spherical particles were utilized to represent both sand and gravel particles, with their grain size distributions designed to be parallel. Given the focus on investigating the particle size effect resulting from the gravel replacement technique, the grain size distributions of the sand particles remained consistent between the model gravelly soils and prototype gravelly soils, while the gravel particles were scaled by a size ratio (*χ*’) of 0.667. The detailed grain size distributions of the DEM models are illustrated in Figure 1. Then, according to the specified distributions, these fine and coarse particles were mixed and deposited into the cylinder in a stratified manner with four layers [35]. The cylinder had dimensions of *ϕ*60 × 120 mm and *ϕ*80 × 160 mm for the model and prototype gravelly soils, respectively. The ratio of the cylinder diameter to the maximum particle size was maintained at 6, ensuring compliance with the requirements of representative elementary volume (REV) [7]. Following particle deposition, all fine and coarse particles experienced a series of processes, including initial size reduction and subsequent enlargement to their original dimensions over three steps. During these processes, cycles were applied to eliminate particle overlaps and to balance the contact forces between particles [22].

The Hertz–Mindlin contact model was employed to simulate particle interactions, capturing the pressure-dependent behaviors of granular soils [35,36,37,38]. The linear contact model was used to depict interactions between walls and particles. The model parameters utilized in this study are listed in Table 2, mainly derived from previous DEM numerical studies [22,35]. It is worth noting that the parameters for both coarse particles and fine particles were maintained identically, aligning with the objective of modeling naturally deposited gravelly soils. In such soils, fine sand particles are often generated from the weathering and degradation of coarse gravel particles due to geological processes, sedimentation, and climatic influences [2,5].

### 3.2. Loading Procedures

**Figure 2:**
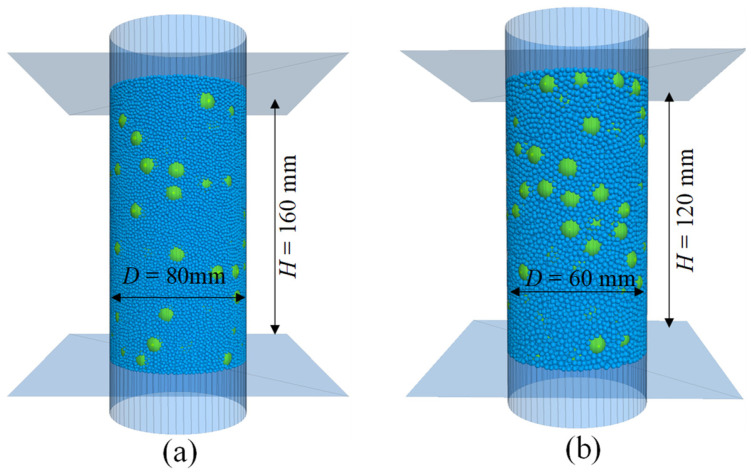
The prepared DEM models after isotropic consolidation for GC40 gravelly soils: (**a**) prototype soils; (**b**) model soils.

Following the initial setup of the DEM model, additional cycles were employed to reduce the unbalanced particle contact forces, i.e., the ratio of the mean unbalanced force to the mean contact force is controlled to below 10^−5^. Subsequently, the prepared DEM model was subjected to isotropic consolidation under specified confining stress levels by using a numerical servo-control mechanism [35]. This process was terminated once the stress tolerance criterion fell below 0.1% [39]. Figure 2 illustrates the typical DEM models for GC40 gravelly soils following the isotropic consolidation process. After consolidation, two distinct loading patterns were employed to achieve the simulation objectives: drained triaxial tests and shear modulus measurements.

The shear modulus was measured by imposing displacement-controlled loading on the cylinder’s top and bottom walls, with synchronized adjustments to lateral walls to ensure cylinder volume consistency. Aligned with standardized protocols for laboratory element testing (e.g., [17,40]), a sinusoidal displacement waveform spanning five cycles was applied. Data from the third cycle, deemed representative under stabilized conditions, were selected to calculate the shear modulus for different strain levels [10].

The drained triaxial tests were simulated by imposing a controlled displacement velocity on the cylinder’s top and bottom walls, while a numerical servo-control mechanism was introduced to maintain constant confining pressure on the lateral walls. To ensure quasi-static shearing conditions, the shear velocity was carefully limited to 0.01 m/s, guided by the inertia parameter (*I*_t_), defined in Equation (7) [41]. This parameter remained below 10^−3^ throughout the simulations, confirming adherence to quasi-static requirements.

(7) $$I_{t}=\dot{\varepsilon }\sqrt{\frac{m_{p}}{p^{\prime }}}=\frac{v}{h_{0}}\sqrt{\frac{m_{p}}{p^{\prime }}}$$

where $\dot{\varepsilon }$ represents the strain rate during axial compression; *m*_p_ denotes the mass of particles; *v* corresponds to the velocity during the shear process; *h*_0_ signifies the initial height of the DEM model; *p*’ is the mean effective pressure acting on the DEM model.

### 3.3. Simulation Arrangement

Simulations were performed for four gravel content (*GC*) values (20%, 40%, 60%, and 80%) and one particle size ratio (*χ*’ = 0.667) to systematically analyze the particle size effect. Small-strain shear modulus tests were conducted under four confining pressures (i.e., 50 kPa, 100 kPa, 200 kPa, and 400 kPa), enabling the determination of the stiffness scaling relation (*λ*_D_) and other related scaling relations. To evaluate the efficacy of the updated scaling relations for correcting the particle size effect, drained triaxial tests and shear modulus reduction tests were simulated under identical void ratios and identical confining pressure of 100 kPa. These simulations provide critical insights into the predictive accuracy of the developed scaling relations across varying mechanical responses.

## 4. Simulation Results and Analysis

### 4.1. Determining the Scaling Relation for Stiffness

**Figure 3:**
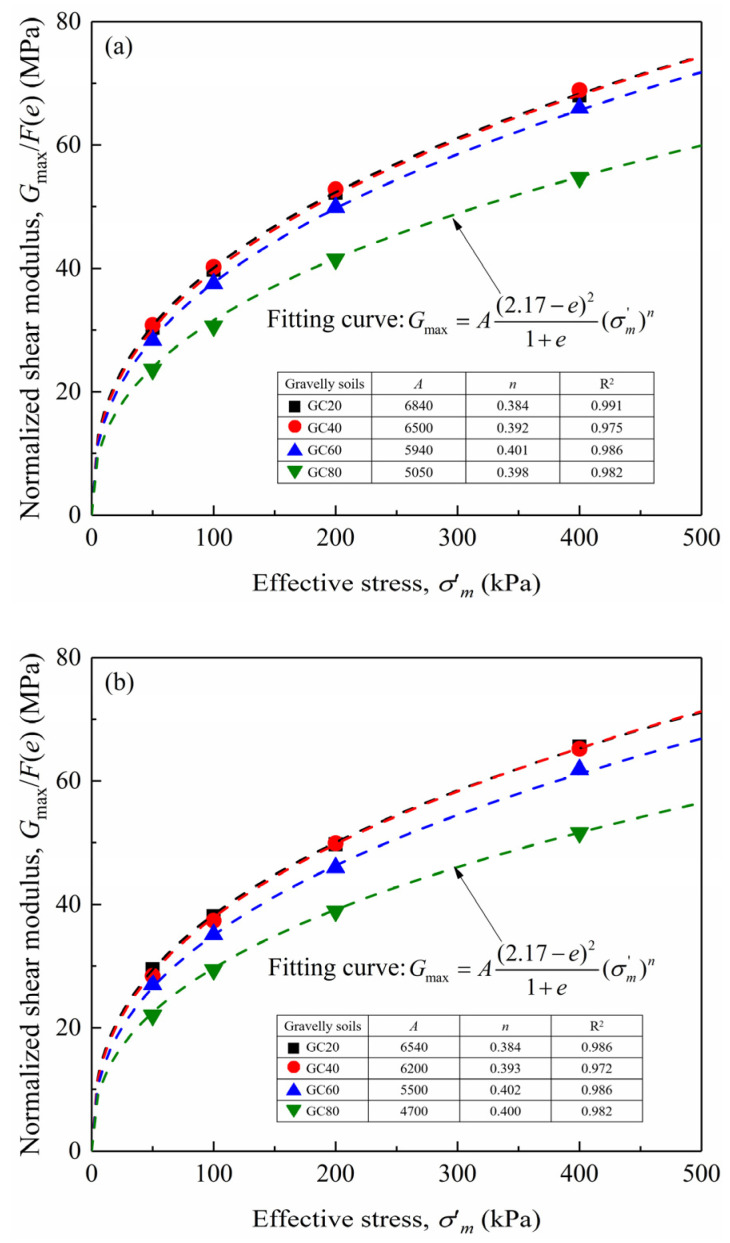
Small-strain shear modulus of the prototype gravelly soils and model gravelly soils: (**a**) prototype gravelly soils; (**b**) model gravelly soils.

**Figure 4:**
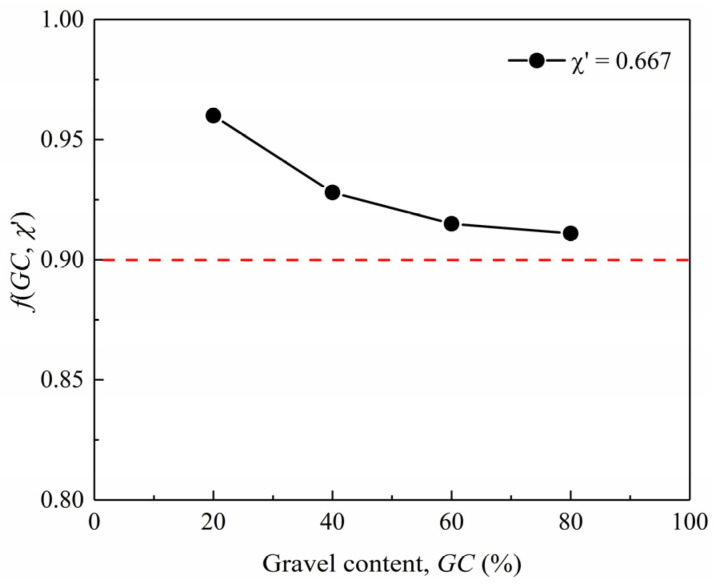
Relationship between *f*(*GC*, *χ*’) and *GC*.

To determine the scaling relations for correcting the particle size effect, small-strain shear modulus tests were initially conducted through simulations. At the low strain level of approximately 10^−6;^, the stress–strain relationship during shear loading exhibits near-linear behavior. The small-strain shear modulus was calculated using the secant modulus at the stress reversal point, as defined by Equation (8), consistent with established laboratory methodologies [17,25,30]. Figure 3 illustrates the calculated small-strain shear modulus for both prototype and model gravelly soils under varying confining pressures, with the corresponding fitting parameters *A* and *n* summarized in Table 3. It can be observed that under identical *GC* and void ratio conditions, prototype gravelly soils exhibit slightly higher *A* values compared to model gravelly soils, while the exponent *n* remains consistent between the two soils. This supports the validity of the assumption *n*_m_ = *n*_p_ in the development of the updated scaling relations. The higher *A* values in prototype gravelly soils indicate greater soil small-strain stiffness, which could be explained by the stiffness reinforcement effect induced by the particle size effect. In other words, the larger gravel particles in the gravelly soils have positive effects on increasing soil stiffness, demonstrating that the gravel replacement technique will lead to a reduction in soil stiffness. The scaling relation for stiffness *λ*_ε_ under the condition of *χ*’ = 0.667 could be calculated according to Equation (5). As shown in Figure 4, *f*(*GC*, *χ*’) slightly decreases with increasing *GC*, which suggests that the gravel replacement technique has more influence on reducing the soil stiffness of gravelly soils with high *GC*s. Even so, the gravel replacement technique has limited influence on the soil stiffness since *f*(*GC*, *χ*’) is generally within the range of 0.9–0.96, covering all *GC* conditions.

(8) $$G=\frac{\Delta q}{2\Delta \gamma }=\frac{\Delta \sigma_{1}-\Delta \sigma_{3}}{2\left(\Delta \varepsilon_{1}-\Delta \varepsilon_{3}\right)}$$

where Δ*q* denotes the deviator stress variation; Δ*γ* represents the shear strain variation; Δ*σ*_1_ and Δ*σ*_3_ correspond to the vertical and lateral effective stress variations, respectively; Δ*ε*_1_ and Δ*ε*_3_ signify the vertical and lateral strain variations, respectively.

### 4.2. Drained Triaxial Test Simulation Results and Analysis

**Figure 5:**
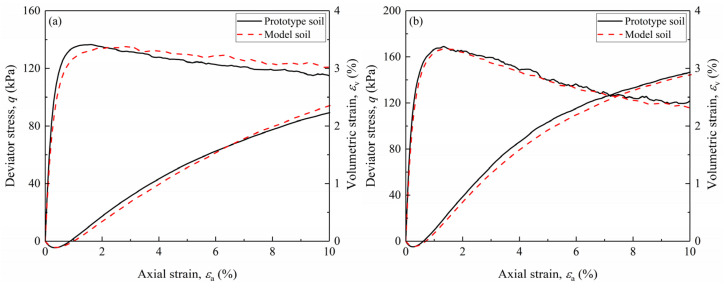

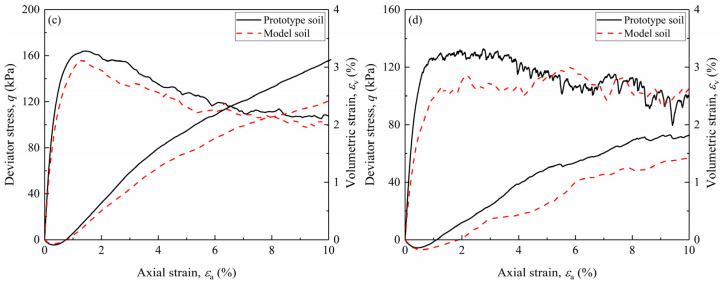
Simulation results of the drained triaxial tests for the prototype gravelly soils and model gravelly soils: (**a**) GC20; (**b**) GC40; (**c**) GC60; (**d**) GC80.

To evaluate the efficacy of the developed scaling relations for correcting the particle size effect, drained triaxial tests were simulated for both prototype and model gravelly soils. Figure 5 presents the shear responses of the DEM models, revealing notable discrepancies in stress–strain relationships and volumetric strain behaviors between the prototype gravelly soils and model gravelly soils during the drained shearing. These differences, amplified with increasing *GC*, are primarily attributable to the particle size effect. Notably, the peak shear strengths of the prototype gravelly soils and model gravelly soils differ by less than 5% under identical void ratio conditions, underscoring the minimal impact of gravel replacement techniques on peak strength. The prototype gravelly soils exhibited a higher secant shear modulus compared to the model gravelly soils, a trend consistent with observations from small-strain shear modulus analysis. This stiffness increase in prototype soils could be explained by the reinforcement effect of the larger gravel particles. However, the dilation responses of the prototype gravelly soils and model gravelly soils were consistent.

Using the scaling relations derived from small-strain shear modulus simulations and the scaling relations outlined in Table 1, the scaling relations for additional mechanical properties were systematically determined. Table 4 summarizes the calculated scaling relations for the four prototype gravelly soils analyzed in this study.

**Figure 6:**
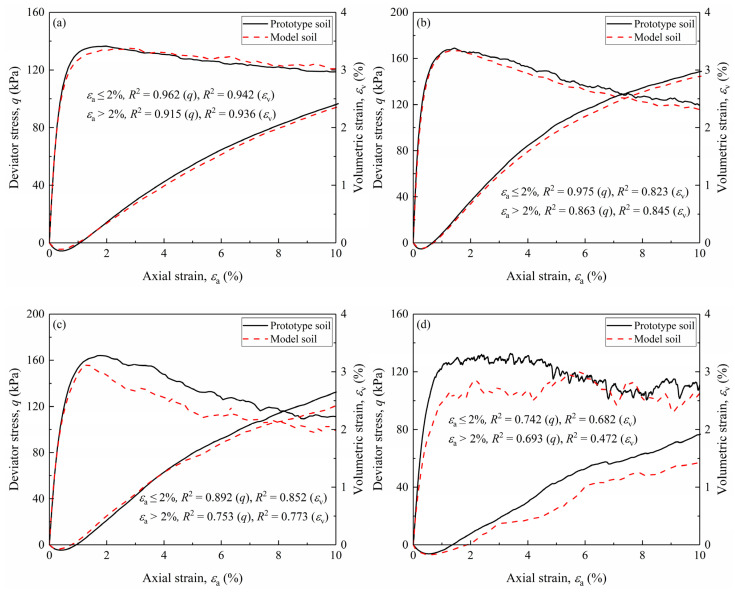
Comparisons between the scaled and prototype shear responses for: (**a**) GC20; (**b**) GC40; (**c**) GC60; (**d**) GC80.

Using the scaling relations outlined in Table 3, the shear stress–strain and volumetric strain behaviors of the model gravelly soils are scaled to simulate the corresponding behaviors of the prototype gravelly soils, as shown in Figure 5. Notably, since the scaling relation for density (*λ*_ρ_) equals 1, the shear stress behaviors of the model gravelly soils remain unchanged. However, shear and volumetric strains are adjusted by multiplying them with the strain scaling relation (*λ*_ε_). Figure 6 compares the shear responses of the scaled model gravelly soil with the direct simulation results of the prototype gravelly soils. It can be observed that the scaling process effectively minimizes discrepancies in both shear strain and volumetric strain responses across small to large strain ranges. For GC20, GC40, and GC60 gravelly soils, the scaled shear responses aligned closely with prototype behaviors within strains up to 2%, reflecting the sand-dominated skeleton structure of gravelly soils with low *GC*s. However, for the GC80 DEM model, the discrepancies in the small shear strains are still large, which could be explained by the changes in the skeleton structure of gravel-dominant gravelly soils with high *GC*s. In general, the above results support the proposed updated scaling relations for correcting the particle size effect, suggesting the high applicability of Rocha’s assumption for soil deformation analysis subjected to small-to-medium strains. However, the moderate alignment in high *GC* cases indicates limitations in using gravel replacement techniques for predicting the prototype behaviors of gravel-dominated gravelly soils with overlarge gravel particles, as skeleton restructuring introduces inherent uncertainties. This underscores the need for cautious application of the developed scaling relations in high *GC* conditions to mitigate prediction errors.

### 4.3. Shear Modulus Test Simulation Results and Analysis

**Figure 7:**
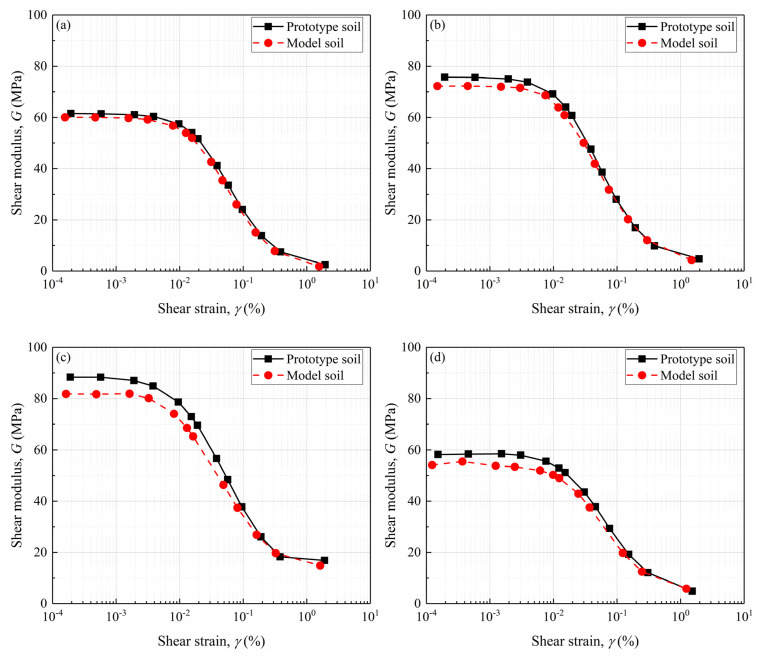
Shear modulus of the prototype and model gravelly soils for: (**a**) GC20; (**b**) GC40; (**c**) GC60; (**d**) GC80.

Using the methodology outlined for small-strain shear modulus measurement, the shear modulus *G* at varying strain levels was calculated following Equation (8). Figure 7 illustrates the variation of *G* with shear strain *γ* (i.e., shear strain at the reversal stress point) for all DEM models. Consistent with the above observations, the prototype gravelly soils exhibit higher shear modulus than the model gravelly soils across all strain levels, which could also be explained by the particle size effect on increasing soil stiffness by increasing interparticle contact forces and improving load transfer efficiency [5,22].

**Figure 8:**
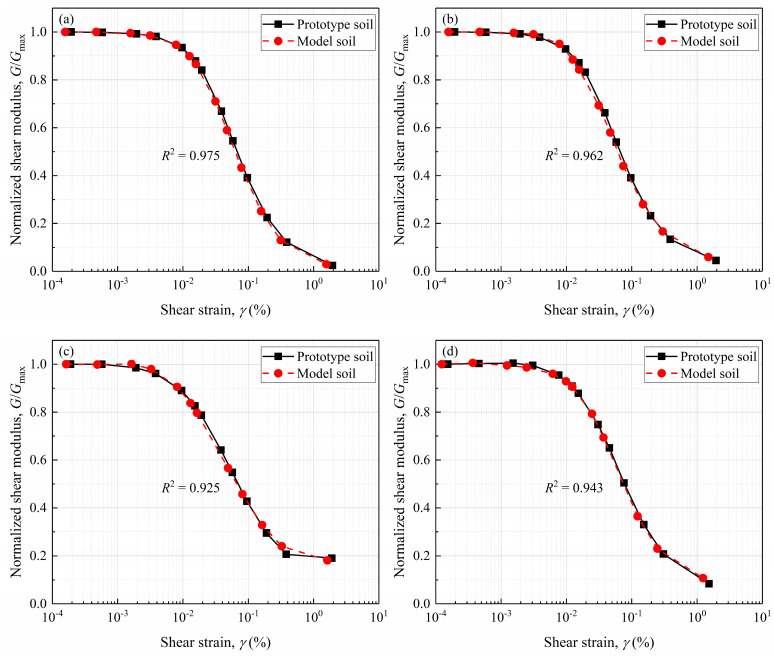
Comparisons between the scaled and prototype shear modulus reduction curves for: (**a**) GC20; (**b**) GC40; (**c**) GC60; (**d**) GC80.

Following the data processing method used in drained shear response correction, Figure 8 compares the scaled and prototype normalized shear modulus (*G*/*G*_max_) reduction curves for all DEM models. In this figure, the shear modulus is normalized by the small-strain shear modulus, and since *G*/*G*_max_ is a dimensionless quantity, no scaling relation is applied to *G*/*G*_max_, while the shear strain is modified by the scaling relation *λ*_ε_. It can be observed that although the particle size effect reduces the shear modulus of the prototype gravelly soils, the impact of such treatment on the normalized shear modulus reduction curves from small to large strains is relatively minor. This phenomenon further underscores the robustness of Rocha’s assumption. The results demonstrate that by acquiring the small-strain shear modulus (or shear wave velocity) of both prototype and model gravelly soils, the dynamic stress–strain behaviors of the prototype gravelly soils can be accurately extrapolated from the model gravelly soil data through inverse analysis. This approach leverages the scaling relationships derived from the particle size effect, ensuring highly accurate predictions of dynamic behaviors across varying *GC*s and strain regimes.

The validation results derived from comparative analysis of shear stress–strain relationships and shear modulus reduction curves support Rocha’s assumption, demonstrating its efficacy in addressing deformation-related problems instead of the stability problem in the ultimate state. The negligible discrepancies observed in shear responses across small to medium strain ranges (*γ* < 1%) further confirm that maintaining identical void ratios is critical for gravel replacement techniques that cause changes in particle sizes. Under these conditions, prototype and model gravelly soils with the same *GC* achieve optimal similarity in shear behavior, enabling the scaling relations to effectively correct the particle size effect. The developed scaling relations thus provide a reliable basis for extrapolating model test data to prototype-scale predictions in geotechnical applications controlled by small to medium strains. Additionally, it should be noted that the drained triaxial test simulations only provide a preliminary validation; other test simulations, such as uniaxial tests, as well as their relationships with the triaxial test results, deserve further studies [42].

## 5. Conclusions

This study systematically investigates the particle size effect on the shear behavior of binary mixtures through a series of DEM simulations of drained shear tests and shear modulus tests, focusing on the implications of gravel replacement techniques for laboratory testing. The updated scaling relations are proposed and validated to correct the particle size effect for gravelly soils with overlarge gravel particles. The main conclusions are summarized as follows:

(1) The simulation results demonstrate that larger gravel particles significantly enhance soil stiffness, while the gravel replacement technique, which substitutes overlarge particles with finer ones, leads to a reduction in soil stiffness from small to large shear strains. This effect is more pronounced in soils with high *GC*s (e.g., *GC* > 60%), although the impact on peak shear strength remains minimal.
(2) The scaling relations for correcting the particle size effect for gravelly soils are proposed based on the well-known Iai’s GSR. The scaling relations for stiffness and strain were successfully applied to predict the mechanical responses of prototype soils from model tests, particularly for small to medium strain levels. This validation confirms the reliability of the updated scaling relations in predicting the shear responses of gravelly soils with overlarge particles.
(3) Rocha’s assumption, which relates stress and strain similarities between model and prototype soils, is validated to be highly applicable for correcting particle size effects in deformation problems. However, for gravel-dominant soils with high *GC*s, this assumption shows moderate applicability, indicating some limitations in accurately predicting shear behavior, especially at large strains.

This study offers geotechnical engineers valuable insights to optimize lab testing of gravelly soils, especially with overlarge particles, and better predict soil behavior using the developed scaling relations, enhancing experimental design and engineering decision-making. Future research should focus on expanding the scaling relations to complex soil types, exploring particle size effects under diverse loading conditions, validating results through large-scale experiments, and developing comprehensive constitutive models that account for particle size, shape, and gradation.

## Figures and Tables

**Table 1:** Iai’s GSR and the developed scaling relations for correcting the particle size effect.

Mechanical Properties	Scaling Relations of the Type I GSR by Iai [25]	The Developed Scaling Relations for Correcting the Particle Size Effect
Length	*λ*	1
Density	*λ*_ρ_	1
Time	(*λλ*_ε_)^0.5^	(1/*f*(GC, *χ′*))^0.5^
Frequency	(*λλ*_ε_)^−0.5^	(1/*f*(GC, *χ′*))^−0.5^
Acceleration	1	1
Velocity	(*λλ*_ε_)^0.5^	(1/*f*(GC, *χ′*))^0.5^
Displacement	*λλ*_ε_	1/*f*(GC, *χ′*)
Stress	*λλ*_ρ_	1
Strain	*λ*_ε_	1/*f*(GC, *χ′*)
Stiffness	*λλ*_ρ_/*λ*_ε_	*f*(GC, *χ′*)
Permeability	(*λλ*_ε_)^0.5^/*λ*_ρ_	(1/*f*(GC, *χ′*))^0.5^
Pore pressure	*λλ*_ρ_	1

**Table 2:** Model parameters used in this study.

Parameters	Value	References
Particle density, *ρ* (g/cm^3^)	2.63	Xia et al. (2024) [22]
Particle shear modulus, *G* (kPa)	10^7^	Xia et al. (2024) [23]
Normal stiffness for wall-particle contacts, *k*_n_ (kN/m)	10^5^	Xu et al. (2015) [35]
Wall-particle frictional coefficient, *μ*_wp_	0	Xia et al. (2024) [22]
Particle frictional coefficient, *μ*	0.5	Gong and Liu (2017) [39]
Particle Poisson’s ratio, *ν*	0.2	Xia et al. (2024) [22]
Damping factor, *α*	0.7	Xu et al. (2015) [35]

**Table 3:** Fitting parameters *A* and *n* for the prototype gravelly soils and model gravelly soils.

GC	*A*_p_	*A*_m_	*n*_p_	*n*_m_
20%	6840	6540	0.384	0.384
40%	6500	6200	0.392	0.393
60%	5940	5500	0.401	0.402
80%	5050	4700	0.398	0.400

**Note: The subscripts p and m represent prototype and model, respectively.**

**Table 4:** The scaling relations for different prototype gravelly soils.

GC	Soil Type	Void Ratio	Shear Modulus (MPa)	*λ*	*λ*_ρ_	*λ*_ε_
20%	Prototype	0.558	62.51	1	1	0.960
	Model	0.559	60.03			
40%	Prototype	0.463	76.71	1	1	0.928
	Model	0.457	71.22			
60%	Prototype	0.419	89.36	1	1	0.915
	Model	0.420	81.81			
80%	Prototype	0.521	58.24	1	1	0.911
	Model	0.436	53.11			

## Data Availability

The original contributions presented in this study are included in the article. Further inquiries can be directed to the corresponding author.
